# Disseminated and ulcerative basidiobolomycosis simulating a Buruli ulcer in an immunocompetent girl in Southern Benin

**DOI:** 10.11604/pamj.2020.37.227.20994

**Published:** 2020-11-11

**Authors:** Akimath Habib, Christelle D´almeida, Bérénice Degboe, Benjamin Morvant, Marlène Lyne Ganlonon, Ambroise Adeye, Anne Croue, Maxime Kiki, Espoir Sodjinou

**Affiliations:** 1 Centre de Dépistage et de Traitement de la Lèpre et de l´Ulcère de Buruli de Pobè, Fondation Raoul Follereau, Cotonou, Bénin,; 2 Service de Dermatologie-Vénérologie, Hôpital d´Instruction des Armées de Cotonou, Cotonou, Bénin,; 3 Faculté des Sciences de la Santé, Université d´Abomey-Calavi, Cotonou, Bénin,; 4 Service de Dermatologie-Vénérologie, Centre National Hospitalier et Universitaire Hubert Koutoukou Maga, Cotonou, Bénin,; 5 Département de Pathologie Cellulaire et Tissulaire, Unité de Formation d'Anatomie Cytologie Pathologique, Centre Hospitalier et Universitaire d´Angers, Angers, France

**Keywords:** Basidiobolomycosis, Buruli ulcer, co-endemicity, mycosis, histopathology

## Abstract

Basidiobolomycosis is a subcutaneous mycosis, for which non-specific clinical presentation can be a source of diagnostic wandering. A 5-year-old girl was brought for consultation with chronic ulcers of the pelvic limbs evolving for 8 months. The lesions started when the girl was 18 months old with a painless, pruritic nodule of the right buttock, indurated placard following progressive extension to the pelvic limbs, back and abdomen, and secondarily ulcerated in several places. On examination, there was an alteration of the general condition, a large, indurated and erythematous plaque, with sharp edges. On this plaque, there were nodular lesions and necrotic ulcers, with detached margins. The left knee was blocked in flexion. Ziehl staining and polymerase chain reaction for Mycobacterium ulcerans were negative. The histopathological picture was suggestive of basidiobolomycosis. The evolution was favorable after giving her ketoconazole (100mg per day) for 14 weeks associated with surgery and physiotherapy. This clinical case confirms the difficulties in diagnosing basidiobolomycosis, especially in endemic areas of Buruli ulcer.

## Introduction

Basidiobolomycosis is a rare deep mycosis, described for the first time in 1956 in Indonesia [1,2]. Due to *Basidiobolus ranarum*, a saprophytic soil and plant agent, basidiobolomycosis occurs in black Africa, India, the Far East, Latin America and Southern United-States of America. It affects children and adolescents, with a maximum of between 6 and 10 years [2]. Inoculation is transcutaneous direct through microtrauma, stings of contaminated spines, insect bites [3]. The clinic is made of firm dermo-hypodermic plaques that are very clearly circumscribed, and generally cold and painless; it becomes hot and painful by outbreaks. These cupboards can ulcerate during evolution [3]. Due to its non-specific clinical features, it raises the problem of differential diagnosis with Buruli ulcer especially in co-endemic areas [2,4-6]. Basidiobolomycosis can cause invasive pathology by lymphatic or vascular dissemination [3,7]. Positive diagnosis is based on histopathology with the presence of the Splendore-Hoeppli phenomenon in the dermis [3,6]. Treatment currently relies on imidazole antifungals: itraconazole or long-term ketoconazole with few side effects [1,3,8]. We report a case of disseminated basidiobolomycosis that simulated a multifocal category 3 Buruli ulcer in an immunocompetent 5-year-old girl.

## Patient and observation

**Figure 1:**
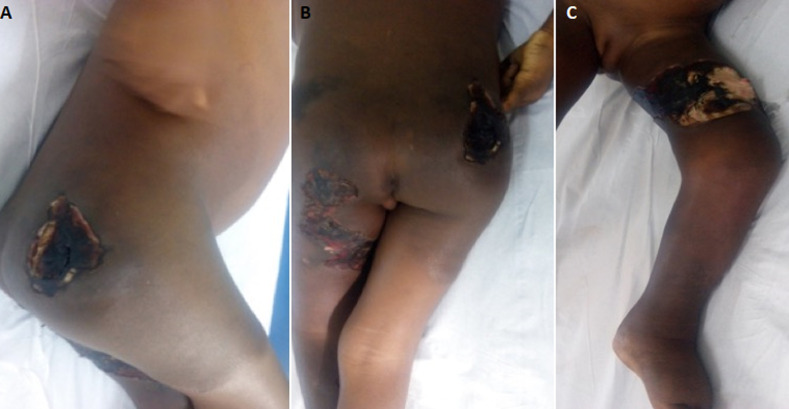
A) erythematous and indurated plaque of lower limbs extending to the abdomen with necrotic ulcer on the right buttock; B) necrotic ulcers on right buttock and posterior side of the left pelvic limb; C) indurated plaque with necrotic ulcer on the inner side of the left thigh associated lymphoedema

A 5-year-old girl living in a rural area was brought for consultation at the center for screening and treatment of leprosy and Buruli ulcer of Pobè, for ulcers of the buttocks that evolved for 8 months. The lesions began 4 years earlier with a painless but itchy nodule of the right buttock, which gradually spread to the pelvic limbs, back and abdomen evolving towards an indurated plaque. This plaque has secondarily ulcerated. The lesions were treated unsuccessfully with unspecified antibiotics and herbal medicine. Vaccination was up to date for the girl´s age and she was not frequenting the river. There was no similar case in her family and entourage. On examination, the patient had a poor general condition, a functional impotence when walking. The palpebral mucous membranes were pale and the bulbar mucous membranes anicterical. There was an infectious syndrome with a fever at 40°C and a steady tachycardia at 100 beats/minute. Her weight was 12 kg for a height of 1 meter (body mass index at 12). The dermatological examination found a large, indurated, erythematous, warm plaques, that was mobile with respect to the deep plane, adhering to the superficial plane. The edges were clearly delineatable by the fingers (Figure 1A), interestingly the lower limbs, the back, and the abdomen. The pubis and the vulva were also indurated. On this large plaque, there were hollow ulcers of variable size (n = 3), with polycyclic contours and fibrinous, necrotic bottom and detached edges (Figure 1B and C). The peri-ulcer area wasn´t pigmented. The back of the left foot was the site of lymphoedema (Figure 1C). There was no damage to the mucous membranes and integuments. The left knee was blocked in flexion causing a functional impotence to walk. Bilateral and inflammatory inguinal lymphadenopathy were noted. The examination of the other organs was normal: no hepatomegaly, no splenomegaly; the pulmonary fields were free. From this symptomatology we evoked diagnostic hypotheses of Buruli ulcer category 3 in the first time.

**Figure 2:**
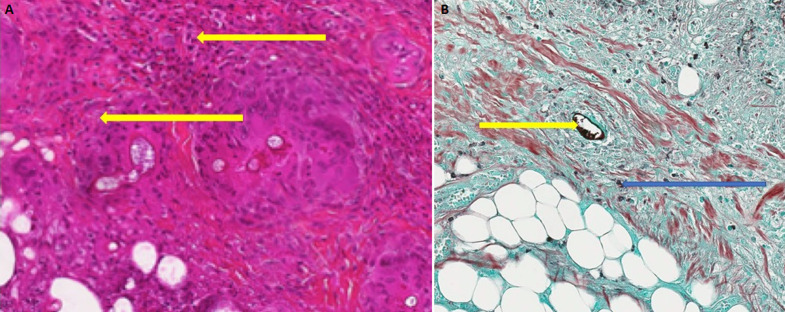
A) hematoxylin-eosin-safran staining showing Splendore-Hoeppli phenomenon (yellow open arrow): perivascular granulomas centered by extracellular eosinophilic (X100); B) grocott staining showing granulomas centered by extracellular eosinophilic masses (blue open arrow) and bulky mycelial filaments (yellow open arrow) (X100)

**Figure 3:**
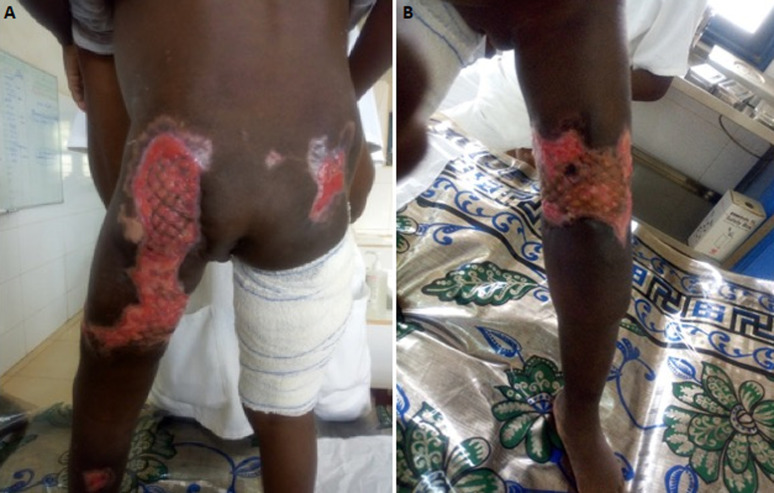
A) ulcers of buttock and posterior face of left lower limb healing after mesh grafting and knee flexion correction; B) ulcers of anterior face of left lower limb healing after mesh grafting

Ziehl Nielsen staining and polymerase chain reaction for *Mycobacterium ulcerans* by gene amplification of a specific genome sequence, IS2404 was negative. Direct examination of a punch biopsy sample shows yeasts and mycelial filaments. Complementary examinations included: hypochromic microcytic anemia, leukocytosis at 19.5 10^9^/L with a predominance of eosinophils at 11% or 2.15 10^9^/L (215/ mm^3^). The C-reactive protein was positive at 192mg/L. Serologies for human immunodeficiency virus (HIV) and hepatitis B infection were negative. In histopathology, the epidermis is well differentiated and discreetly orthokeratotic. The dermis is the site of significant inflammatory fibrosis. Inflammation is polymorphic with numerous eosinophilic polynuclear cells associated with epithelioid granulomas and giganto-cellular granulomas. The granulomas are centered by extracellular eosinophilic masses and bulky mycelial filaments (Splendore-Hoeppli phenomenon), which led to basidiobolomycosis (Figure 2A and B). From these results, the diagnosis of ulcerated disseminated basidiobolomycosis in an immunocompetent girl was retained. The mycological culture and polymerase chain reaction to identify the germ weren´t done because of technical reasons. The patient was put on ketoconazole (100 mg per day) combined with a correction of anemia using oral iron suspension of 5 mg per day and her nutrititional status by enriched porridges and a high protein diet. Transaminase assay was done at the start of the treatment and then every two weeks for monitoring. Surgical management was carried out, consisting initially of a detachment of ulcers, then secondarily a cutaneous mesh transplant to promote the healing of large ulcers. The correction of the flexum, the recovery of the extremities of limbs and walking, as well as the management of lymphoedema were carried out by the physiotherapists. Progression was favorable after 14 weeks of complete medical treatment, lymphoedema, flexum correction, and ulcer healing (Figure 3A and B). There was no recidivism after 6 months and then one year of follow-up.

## Discussion

Basidiobolomycosis is the most common entomophtoromycosis. It is endemic in the tropical and subtropical regions of the world [3,9]. In many of these areas, there is a notion of co-endemicity with Buruli ulcer, as is the case in Southern Benin. Indeed, Atadokpèdé *et al*. as well as Brun *et al*. reported cases of basidiobolomycosis in Benin in areas known to be endemic to Buruli ulcer [5,8]. Although culture wasn´t performed, our case was classified as a basidiobolomycosis based on epidemiological, clinical, biological and histopathological arguments and the efficacy of antifungal drug administered. These arguments helped us also to eliminate mucormycosis, phythiosis or histoplasmosis. Also, there is no reported history of preexisting wound that got worse with pyogenic infectious agent. But by its clinical presentation, it also raises the problem of differential diagnosis with Buruli ulcer in the pre-ulcerative phase as well as in the ulcerative phase [10,11]. Like Buruli ulcer, basidiobolomycosis most commonly affects children between 4-10 years of age [3,10]. This can lead to a diagnosis delay and the evolution can sometimes be fatal [5,12]. In these cases, the histopathology makes it possible to make the diagnosis. Basidiobolomycosis is characterized by an eosinophilic infiltrate that produces a Splendore-Hoeppli phenomenon while the histopathological picture of Buruli ulcer is necrotizing panniculitis with little infiltrate [10,11].

Our clinical case presents two particularities. It is on the one hand a multifocal attack and on the other hand a rare presentation, the ulcerated form. The onset of symptomatology is at the age of 18 months, the age when children have contact with the soil frequently since they are often in a sitting position. The lesions started on the right buttock with progressive spread to the right limb, pubic area, abdomen and contralateral limb. Indeed, the evolution of basidiobolomycosis goes through three stages depending on the competence of the host and the virulence of the fungi. Initially, the infection is confined to the skin or subcutaneous tissues, then depending on the immunity of the patient, the infection can pervade the muscle, tendon, bone, to give a deep extension [7,13]. It can spread through the blood and lymphatic vessels [3]. Our patient had a disseminated form but is HIV-negative. The extensive ulcers and the infectious syndrome contributed to the alteration of the general condition of the patient.

These disseminated forms are not easily diagnosed because the clinical picture doesn´t look like a fungal infection a priori [14]. Early diagnosis is imperative because there is a risk of mortality from spreading the infection [12,14,15]. Digestive disorders during basidiobolomycosis have been reported in the literature and are most often post-mortem diagnosis [14]. Our patient had a disseminated but limited form to the skin without bone or visceral involvement, which probably improved her prognosis. Dissemination in our patient was probably lymphatic, which may also explain the presence of lymphoedema. The absence of an immune deficiency could also help to limit the spread of blood. The ulcerated forms of basidiobolomycosis exist and have been reported by other authors in the literature [14,16]. Saka *et al*. reported in Togo an ulcerated form in a 5-year-old boy simulating a Buruli ulcer [17]. In India, Rajan *et al*. reported a case of basidiobolomycosis in a 20-months-old boy simulating Fournier's gangrene [13]. These clinical cases may suggest the virulent power of the germ that leads to invasion and destruction of the skin tissue, or even systemic invasion. The treatment is medical and relies on imidazole antifungals such as ketoconazole or itraconazole. It is a long treatment but with few side effects [3,18]. In ulcerated forms, surgery is associated [16,13].

## Conclusion

Our clinical case confirms, on the one hand, the diagnostic difficulty of basidiobolomycosis, especially in areas of co-endemicity with Buruli ulcer. On the other hand, it draws attention to the possibility of rare clinical forms such as disseminated and ulcerated forms. Histopathology is a great contribution to an adequate and not mutilating care.
